# RNA-binding proteins modulate drug sensitivity of cancer cells

**DOI:** 10.1042/ETLS20210193

**Published:** 2021-07-30

**Authors:** Oliver Rogoyski, André P. Gerber

**Affiliations:** Department of Microbial Sciences, School of Biosciences and Medicine, Faculty of Health and Medical Sciences, University of Surrey, Guildford, Surrey GU2 7XH, U.K.

**Keywords:** chemotherapy resistance, interactomics, posttranscriptional regulation, RNA-binding proteins

## Abstract

As our understanding of the complex network of regulatory pathways for gene expression continues to grow, avenues of investigation for how these new findings can be utilised in therapeutics are emerging. The recent growth of interest in the RNA binding protein (RBP) interactome has revealed it to be rich in targets linked to, and causative of diseases. While this is, in and of itself, very interesting, evidence is also beginning to arise for how the RBP interactome can act to modulate the response of diseases to existing therapeutic treatments, especially in cancers. Here we highlight this topic, providing examples of work that exemplifies such modulation of chemotherapeutic sensitivity.

## Introduction

Gene expression must be tightly controlled to maintain proper cellular functions. Post-transcriptionally, RNA-binding proteins (RBPs) interact with RNAs in a combinatorial fashion, forming so-called ribonucleoprotein complexes [1–4]. Considering mRNAs, RBPs coordinate all steps towards mRNA templated synthesis of proteins in the cytoplasm. This includes the processing of mRNA-precursors (pre-mRNAs) in the nucleus, followed by export of the spliced, capped and polyadenylated mature mRNAs to the cytoplasm for translation or storage and eventual decay [1,2]. The fate of an mRNA is thereby determined by the dynamic association of many RBPs and non-coding RNAs (ncRNAs), such as microRNAs (miRNAs) and long ncRNAs (lncRNAs) [1,3,5].

Given the crucial roles that mRNA-RBP interactions (alongside various ncRNAs) play in maintaining proper levels, timing and localisation of gene products, there can be dire consequences when this process is perturbed [6]. The disruption of this careful balance can lead to aberrant gene expression, which in turn can contribute to prominent complex diseases such as cancer [6–9]. Mutations in RBPs have also been directly linked to a variety of inherited human diseases, which includes orphan diseases [6], such as mutations in fragile X mental retardation protein (FMRP) leading to fragile X-associated tremor/ataxia syndrome (FXTAS) [10], and muscleblind-like 1 (MBNL1) in myotonic dystrophy type 1 [11]. Interestingly, the top Mendelian disorders affected by RBP mutations tend to be diseases of the nervous system, pathologies of the metabolism, and cell proliferation disorders including certain cancers [6]. Interestingly, there is a strong prevalence for mutations in RNA binding domains (RBDs) that are hallmarks of ‘classical' RBPs and establish specific interactions with RNA targets [6,12].

Considering the importance of RNA–protein interactions in cells, there has been a widespread surge in the examination and analysis of the RBP ‘interactome' at a global level. Besides protein-centred approaches, aimed at identification of RNAs interacting with particular RBPs, a variety of RNA-centred techniques have recently emerged for capturing and examination of the interactome on entire RNA ‘populations' — such as RNA interactome capture (RIC) (reviewed in [4,13]). As such, an increasing number of sophisticated datasets across different organisms, and in different conditions, have created a rich resource for further investigation. Besides many canonical RBPs that bear one or several well-characterised RBDs, many unconventional RBPs, such as metabolic enzymes, heat shock factors, and kinases have been found to interact with RNA in all species examined, and whose functions remain to be discovered [4,14].

Whilst the above approaches can provide a picture of composition and dynamics of the entire RNA-binding proteome (RBPome), more specific approaches for the investigation of the cellular interactome of individual RNAs — by tagging the RNA of interest with an affinity aptamer and recovering with a high-affinity ligand coupled to beads or capture of native RNAs with modified ASOs) — have also come along recently (reviewed in [13]). With these techniques now overcoming many of the previous limitations for detection of specific RNA–RBP interactions (such as efficiency and specificity of RNA capture), novel discoveries have not only been made for mRNA and ncRNAs [13], but also viral-RBP interaction dynamics (e.g. SARS-CoV-2 [15,16]).

The combined use of RNA-centred alongside with protein-centric approaches has and will further allow for identification of RNA–RBP networks that could composite crucial regulatory nodes in gene expression. With a considerable knowledge of how, when, and where in cells these interactions occur, they may become promising targets for therapeutic intervention, especially for diseases arising from the consequences of aberrant gene expression.

## RNA-binding proteins modulating drug sensitivity

Many conventional small molecule drugs have been used for decades for effective cancer treatment, often leading to drug resistance after prolonged application. One such drug is cisplatin (CP) for cancer treatment. CP binds DNA forming intra- and inter-strand DNA cross-links and mono adducts, thereby interfering with transcription and replication, eventually leading to cell toxicity and apoptosis [17]. Despite extensive and effective use of the drug in cancer treatment since the 1960s, development of CP resistance in cancers is relatively commonplace, and provides one of the major limitations of its use. Taking CP resistance as a specific example of a drug facing the challenges of chemoresistance, several global studies investigating changes in the transcriptome or the proteome found an association between CP resistance and aberrant levels and possibly activity of ribonucleoprotein complexes and ribosomes (e.g. [18]), Furthermore, certain RBPs that modulate the sensitivity of cancer cells to CP and other cytostatic drugs inhibiting cell division have been identified, albeit without knowing the underlying targets and functions. For example, heterogenous nuclear ribonucleoprotein C (hnRNPC) was identified as a candidate biomarker for CP, 5′fluorouracil (5-FU), and paclitaxel resistance in gastric cancer cells [19]; the down-regulation of the cold-shock proteins Rbm3 and Cirbp1 impaired survival of prostate cancer and increased the sensitivity to CP and doxorubicin [20], while the knock-down of Cirbp1 decreased proliferation of renal carcinoma cells enhancing sensitivity for gemcitabine [21]; and overexpression of circular RNA La-related RNA binding protein 4 (circ-LARP4) increased the sensitivity of MCF7 breast cancer cells to doxorubicin [22].

Thus, while the importance of RBP mediated post-transcriptional control of gene expression for modulation of drug sensitivity and resistance is becoming eminent [23–25], recent work has also begun to unravel particular RBP–RNA interactions that could contribute to cancer progression and chemotherapy resistance. A recent study by Iadevaia et al. investigated the dynamic RNA–RBP interactions in HEK293 cells treated with CP [26]. Specifically, the work focused on RBP interactions with the *p27/CDKN1B* mRNA, coding for an important tumour suppressor. The stability of this mRNAs was shown to be increased shortly after CP treatment of cells, which could be inferred through elements located in the 3′ untranslated regions (UTR) in the mRNA. Looking at the arrangement of RBPs in the presence of CP as compared with untreated control cells by applying an RNA-centred tandem RNA affinity isolation procedure (tobTRIP), 54 proteins were identified to interact with a GFP-tagged reporter bearing the 3′UTR of the *p27* mRNA. Subsequent knockdown of several of the identified RBPs impeded CP induced expression of *p27* mRNA in HEK293 cells, possibly modulating mRNA stability. Finally, knockdown of KH-type splicing regulatory protein (KHSRP) in MCF7 cells, which are rather resistant to CP treatment, rendered these cells sensitive to CP treatment. Besides highlighting the importance of a post-transcriptional regulatory networks in modulating drug sensitivity, the study demonstrated first use of an RNA-centric approach — applying a mRNA–protein interactome capture technique — to unravel critical factors involved.

Work by Hopkins et al. [27] examined the role of the RBP LARP1 and its interactome in the survival, drug-resistance, and tumorigenesis of epithelial ovarian cancer cells (EOCs), a malignancy with a high mortality rate due to high levels of chemotherapy resistance. LARP1 is a known regulator of both mRNA stability and translation, acting through the mTOR pathway by binding to RAPTOR (which is part of a signalling pathway that regulates cell growth, sensitive to nutrient and insulin levels) [27]. LARP1 was previously known to be highly expressed in hepatocellular and lung cancers, is a predictor of adverse outcomes, and it contributes to cancer progression [28]. LARP1 knockdown in ovarian cancer derived cell lines (SKOV3 and OVCAR8, both highly resistant to platinum-based therapies), decreased cell viability while increasing apoptosis, and enhanced the sensitivity of cells to commonly used drugs for EOC treatment (CP, Paclitaxel and Gemcitabine). This was further confirmed in cells derived from patients that developed platinum-based therapy resistance [27]. Further applying a protein-centred approach (i.e. co-immunoprecipitation of protein and identification of interacting RNAs) revealed that LARP1 was associated with anti- (*BCL2*) and pro-apoptotic (*BIK*) protein-coding mRNAs [27]. LARP1 depletion destabilised *BCL2* transcripts, while stabilising *BIK*, which is in line with an overall increased apoptosis with LARP1 depletion. Knockdown of BCL2 mimicked the effects of LARP1 knockdown, including sensitivity to chemotherapeutics, whilst *BCL2* overexpression rescued the increase in apoptosis caused by LARP1 depletion. Interestingly, parallel work by Heise et al. [29] likewise showed that the paralogous cancer-associated La protein contributes to CP resistance in head and neck squamous cell carcinoma cells by stimulating the translation of BCL2. Thus, both paralogous RBPs are thought to modulate chemosensitivity of cancers by regulating the expression of a common anti-apoptotic mRNA target.

Two further recent examples that showcase RNA-RBP interactions directly influencing survivability and drug sensitivity in cancer cells concern the zinc finger protein ZFP36, whose overexpression inhibited the growth of hepatocellular carcinoma (HCC) tumour cells and promoted 5-FU sensitivity in a xenograft tumour mice model [30]; and CELF2, which inhibits ovarian cancer progression and increases sensitivity to CP [31]. ZFP36 was shown to bind to the 3′UTR of *PRC1* mRNA, thereby down-regulating PRC1 expression and exerting an anti-tumour effect. From this, the authors postulate a new therapeutic strategy that could be explored by exploiting the ZFP36/Prc1 axis [30]. CELF2 was found to bind to and stabilise *FAM198B* mRNAs, suggesting an CELF2/FAM198B axis as a therapeutic target for ovarian cancer treatment [31].

Besides these exemplary studies, the information contained in various public databases provides a complementary and useful resource to predict and eventually establish links between RBPs, cancer outcome and sensitivity to a range of chemotherapeutics. For example, Gao et al. [32] considered The Cancer Genome Atlas (TGCA) database to identify RBPs differentially expressed in prostate cancer patients with poor prognosis, designating those RBP profiles as ‘high risk'. Calculating the likelihood of response to the chemotherapeutic drug bicalutamide and CP based on data extracted from the Genomics of Drug Sensitivity in Cancer database (https://www.cancerrxgene.org/), it was suggested that high risk RBP profiles are likely to have a weaker response to these drugs. Likewise, considering the GTEx dataset [33], Kosti et al. [34] found that the chemotherapeutic drug temozolomide saw a better response in glioblastoma patients with low levels of the RBP, SERBP1, which was found to be a critical player in cancer metabolism and for epigenetic features. The promising nature seen in screening data, such as these, once more confirms the value of further exploring the impact of RBPs on sensitivity to chemotherapeutics.

## Conclusions

Recent data suggests that drugs designed to impede the functions of pro-survival RBPs, possibly in combination with conventional chemotherapeutics, could be a promising therapeutic intervention in the treatment of chemotherapy-resistant cancers (although knock-on effects on mRNA targets must also be considered). Thereby, the mechanisms by which RBP interactions may impact cell survival and growth do not have to have evolved to be specific to their role in chemoresistance. RBPs have been shown to play roles in the signalling pathways, regulation of RNAs, and protein interactions that impact crucial factors in cancer progression and survivability (malignant cell growth, metastasis, and cell proliferation). As well as this, many RBPs are also involved in DNA damage response and DNA repair pathways [35,36], which could establish potential connections for directly impacting the fate of cells exposed to DNA targeting chemotherapeutics such as CP.

While considered non-druggable for a long time [37], largely due to being devoid of well-defined binding pockets characteristic of many enzymes, RNA–protein interactions can now be specifically targeted by small molecules or ASOs that block access of RBPs to their cognate binding sites. While several such drugs are currently in clinical trials or already made it to the clinics, research in the forthcoming years will certainly reveal many more molecules that can specifically interfere with RNA–protein interactions and which can then be exploited for the treatment of disease [38,39]. In this area, fundamental studies considering the modulation of drug sensitivity by RBPs and their interactions could provide novel therapeutic targets for the treatment of chemotherapy resistant cancers, bearing potential for exciting advances.

## Open Access

Open access for this article was enabled by the participation of University of Surrey in an all-inclusive *Read & Publish* pilot with Portland Press and the Biochemical Society under a transformative agreement with JISC.

## Abbreviations

5-FU: 5′fluorouracil
CP: cisplatin
RBDs: RNA binding domains
RBP: RNA binding protein
UTR: untranslated regions
